# Environmentally Friendly Nanoporous Polymeric Gels for Sustainable Wastewater Treatment

**DOI:** 10.3390/gels10120756

**Published:** 2024-11-22

**Authors:** Tarek M. Madkour, Rasha E. Elsayed, Rasha A. Azzam

**Affiliations:** 1 Department of Chemistry, School of Science and Engineering, The American University in Cairo, Cairo 11835, Egypt; r.essam@aucegypt.edu; 2 Department of Chemistry, Helwan University, Ain-Helwan 11795, Egypt; rasha_azzam@science.helwan.edu.eg

**Keywords:** adsorption, bio-based polymers, catalytic nickel nanoparticles, toxic organic pollutants, industrial wastewater treatment

## Abstract

Environmentally friendly nanoporous gels are tailor-designed and employed in the adsorption of toxic organic pollutants in wastewater. To ensure the maximum adsorption of the contaminant molecules by the gels, molecular modeling techniques were used to evaluate the binding affinity between the toxic organic contaminants such as methylene blue (MB) and Congo red (CR) and various biopolymers. To generate nanopores in the matrix of the polymeric gels, salt crystals were used as porogen. The pores were then used to accommodate catalytic nickel (Ni^0^) nanoparticles. Under UV irradiation, the nanoparticles demonstrated the effective adsorption and photocatalytic degradation of both the methylene blue and Congo red dyes, achieving removal efficiencies of up to 90% for MB and 80% for CR. The thermodynamic analysis suggested a spontaneous endothermic dissociative adsorption mechanism, which implies the oxidative catalytic degradation of the dyes. The kinetic modeling suggested a pseudo-second-order model, while the model for intra-particle diffusion revealed that Congo red diffuses faster than methylene blue. MB adsorption followed a Langmuir isotherm, while CR adsorption followed a linear isotherm. The results confirm that dye molecules initially undergo physisorption and subsequent dissociative adsorption. The products of the catalytic degradation of methylene blue continue to be absorbed on the surface of the nanoparticles, while those of Congo red switch to preferential desorption.

## 1. Introduction

The release of hazardous substances into Earth’s ecosystem presents considerable dangers to both the environment and human health. Industrial effluents, particularly those containing toxic dyes, constitute a major source of water pollution, adversely affecting both human health and aquatic ecosystems [1]. The pharmaceutical, textile, and chemical industries are the primary contributors to dye production, generating an estimated 700,000 tons annually [2,3]. Some soluble organic dyes are resistant to degradation and exhibit inherent toxicity. Their persistent nature complicates removal through conventional treatment techniques. Additionally, these dyes obstruct light penetration in aquatic environments, which hinders photosynthesis and elevates the demand for dissolved oxygen, ultimately leading to detrimental effects on aquatic life [3,4,5,6].

A variety of strategies have been implemented to remove organic dyes from wastewater through chemical, biological, and physical remediation methods. These approaches include chemical precipitation [7], ion exchange [8], sonochemical treatment [9], adsorption [10], electrochemical processes, degradation [11], and ozonation [12]. Among these, photocatalytic degradation has emerged as a particularly effective technique for dye removal from wastewater, since it offers a promising solution for environmental remediation due to its environmental friendliness with mild reaction conditions and no harmful byproducts, as well as high efficiency, versatility, and evident cost effectiveness. During photocatalytic degradation, the catalytic particles absorb ultraviolet (UV) light, which induces an electronic transition that generates electron–hole pairs and splits the water molecules into radicals causing the dye molecules to break down [13,14]. As previously mentioned, the presence of methylene blue (MB) in water sources presents significant health risks, including the potential to cause nausea, vomiting, and tissue necrosis [15]. Kistan et al. [16] demonstrated the effectiveness of zinc oxide (ZnO) nanoparticles as a photocatalyst for degrading MB under UV light. More recently, Ghiyasiyan-Arani et al. [17] synthesized different cobalt vanadate nanostructures (Co_2_V_2_O_7_, CoV_2_O_6_, and Co_3_V_2_O_8_) using a simple solid-state method for the disintegration of methylene blue dye. Additionally, Congo red is also extensively used in industries such as plastics, rubber, printing, and dyeing [18]. However, the conversion of CR to benzidine is a global concern due to the carcinogenic and mutagenic properties of benzidine [19]. Significant research has been directed toward developing effective catalytic nanocomposites for the degradation of CR dye. Liu et al. [20]. synthesized a TiO_2_/WO_3_ nanocomposite that degrades a variety of organic dyes, including Congo red. Tammina et al. [21] also examined the photocatalytic activity of SnO_2_ nanoparticles of three different sizes for the degradation of CR dye under UV light. Din et al. [22] developed catalytic nanoparticles using a green method, rich in phenolic compounds and antioxidants, that was utilized in metal ion reduction. However, the difficulty in separating these nanoparticles from the effluent posed a challenge to the broader application of this promising method. To overcome such an obstacle, we incorporated the nickel nanoparticles into the porous gels, which would enhance the degradation and removal of harmful contaminants from wastewater.

The gels used throughout this study were based on a bio-based polylactic acid (PLA) derived from natural sources, biocompatible polyurethane (PU), and biodegradable polyethylene oxide (PEO), all of which were examined using molecular modeling techniques for the maximum affinity to MB and CR and are readily available and cost-effective. The salt-leaching technique was used to prepare the nanoporous gels, which were then allowed to soak in a nickel salt solution to incorporate the nickel ions into the nanopores and were later reduced into nickel nanoparticles to yield the in situ impregnated sustainable polymeric gels. To our knowledge, the concept of creating nanoporous bio-based gels, followed by the subsequent in situ impregnation with nickel nanoparticles, is a new approach developed by our research team. The gels were thus utilized for the treatment of wastewater containing CR and MB toxic dyes through their degradation under the influence of photocatalysts. This novel approach has been successfully used to achieve up to a 90% removal efficiency of MB and an 80% removal efficiency of CR.

## 2. Results and Discussion

### 2.1. Modeling of the Polymeric Blends

**Figure 1:**
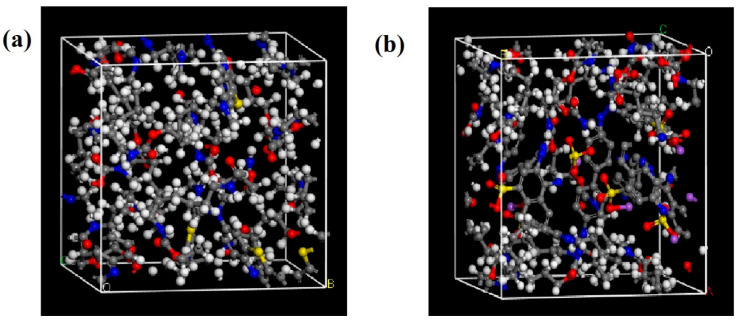
Snapshots of the molecular simulations of (**a**) the polymer blend mixed with MB dye molecules and (**b**) the polymer blend mixed with CR dye molecules. Each cell has a triclinic lattice with a side length of 19.7064 Å and an angle of 90°.

Molecular models of blends of bio-based polylactic acid (PLA), biocompatible polyurethane (PU), and biodegradable polyethylene oxide (PEO) were created using Materials Studio^®^ simulation package, to estimate the binding affinity of MB and CR to the polymer matrix as an expression of the ability of the polymeric gels to adsorb and retain the pollutants [5]. The geometry optimization and molecular dynamics (MD) simulations were run using the COMPASS forcefield to assign the various charges, the Anderson thermostat to control the temperature, and the Ewald summation method. Following the geometry optimization of the simulated models, MD runs were performed at room temperature using NVT for 25 ns with a 1 fs time-step. The trajectories were saved for analysis, and one snapshot was taken every 10,000 steps [23]. Figure 1 shows snapshots of the simulation cells of poly(lactic acid) and the polyurethane blend mixed with MB or CR molecules.

To evaluate the interfacial interaction between the polymeric chains and the contaminant molecules, the binding affinity of the polymeric chains and the dye molecules while implementing the periodic boundary conditions are calculated according to the following: *ϕ*

Δ*H_mix_/V = ϕ*_1_*CED*_1_ + *ϕ*_2_*CED*_2_ − (*ϕ*_1_
*+ ϕ*_2_)*CED*_12_ (1)

where Δ*_i_* is the volume fraction of *i*, and *CED_i_* is its cohesive energy, which is the energy required to break the intermolecular interactions between molecules [24].

**Figure 2:**
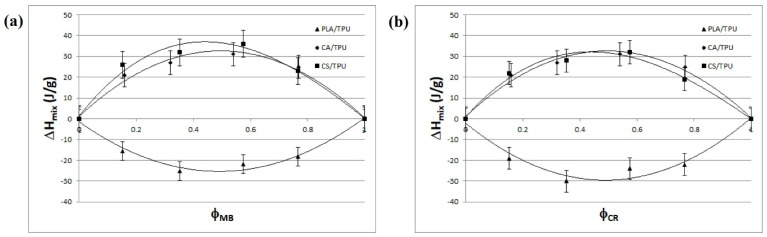
Enthalpy of mixing MB (**a**) and CR (**b**) with the different polymeric blends at different dye mole fractions.

To select the polymeric blend system that has the greatest interfacial interactions with the dyes, values for the enthalpy of mixing of the various systems mixed with MB and CR are plotted in Figure 2.

It is clear from the figures that the poly(lactic acid) blend with thermoplastic polyurethane [25] had the most favorable interactions with the organic dyes, as evidenced by the negative values for the enthalpy of mixing the blend and the dyes at different mole fractions. This observation might be interpreted on the basis that in the case of the other two blends, both cellulose acetate and chitosan are quite hydrophilic and would therefore have unfavorable interactions with the mildly hydrophobic polyurethane chains [26,27].

### 2.2. Characterization of the Physicochemical Characteristics of the Gels

**Figure 3:**
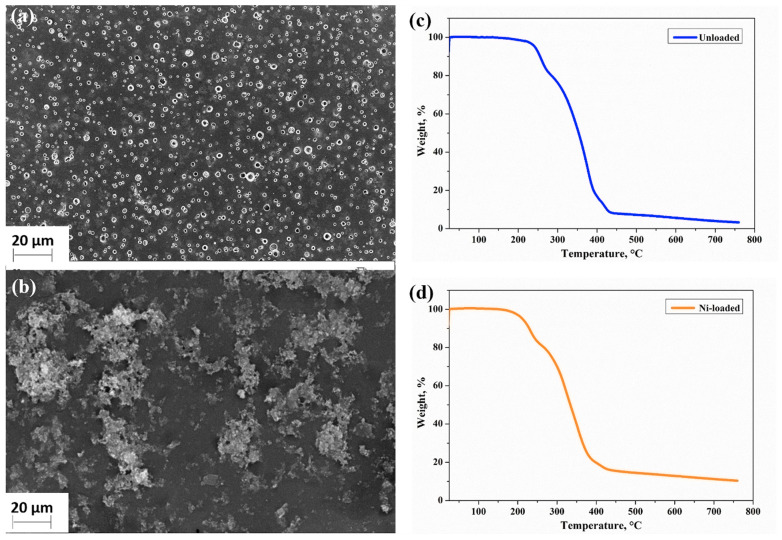
(**a**) SEM image for the unloaded gel, (**b**) SEM image for the Ni-loaded gel, (**c**) TGA for the unloaded gel, and (**d**) TGA for the Ni-loaded gel.

The scanning electron microscopy (SEM) images (Figure 3a,b) illustrated that the unloaded gel (Figure 3a) displayed a porous structure, whereas the Ni-loaded gels (Figure 3b) exhibited a rougher surface [28,29]. The thermogravimetric analysis (TGA) of the unloaded and Ni-loaded gels (Figure 3c,d) revealed a two-step degradation process, characterized by a distinct inflection point in the degradation profile. This inflection is attributed to the breakdown of ester groups in polylactic acid (PLA) and urethane linkages in polyurethane [30,31]. Importantly, the presence of the impregnated Ni^0^ nanometals did not affect the decomposition temperatures during thermal degradation, indicating that the NPs did not catalyze the degradation of the gel network.

FTIR demonstrated the impact of the Ni^0^ impregnation of the gels, shown in Figure S1. The intensity of the 3100 cm^−1^ and 2900 cm^−1^ peaks associated with the C-H alkene and alkane was higher in the impregnated gels compared to the unloaded ones [26].

**Figure 4:**
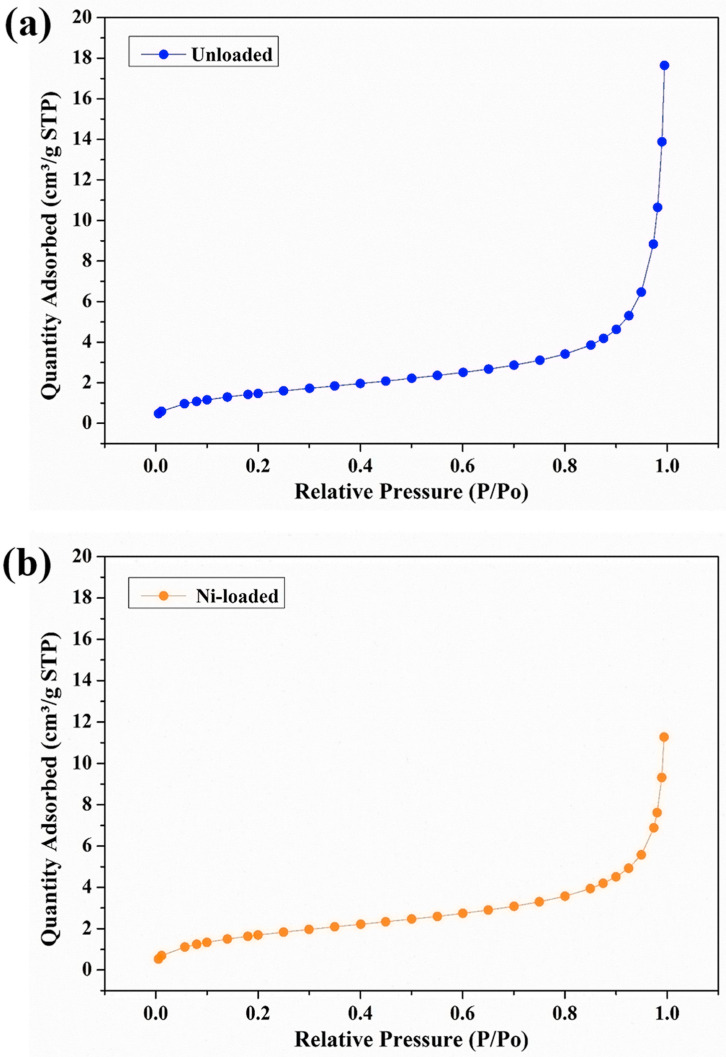
BET isotherms of the unloaded gel (**a**) and the Ni^0^-loaded gel (**b**).

The Brunauer–Emmet–Teller (BET) isotherms for both the unloaded and Ni^0^-loaded gels, shown in Figure 4, were classified as Type IV isotherms, indicating significant adsorption on mesoporous materials. The unloaded gels contained some macropores. The BET results analysis showed that incorporating nanoparticles into the gels increased the pore size of the unloaded gel from 6.69405 nm to 9.77334 nm for the Ni^0^-loaded gel and increased the surface area from 5.9796 m^2^/g to 8.4885 m^2^/g, respectively, due to the added surface area of the metallic nanoparticles in addition to the accessible surface area of the pores within the porous gels. Table S1 provides a comparison of the surface area and pore size of the prepared unloaded and Ni^0^-loaded gels with other bio-based polymer gels from previous studies. The table shows that the surface area and pore size of the prepared gels are lower than those published previously. These low values are important for the gel impregnation with the Ni^0^ nanoparticles, since small pores will trap the nanoparticles within the pore walls and will not allow them to escape into the treated water.

Correlating the results of Figure 3 and Figure 4 provides a clear understanding of the impact of loading Ni nanoparticles on the morphology of the gels [32]. Figure 3a shows a smooth surface with spherical pores, indicating that the material is porous, while Figure 3b shows a rough surface with numerous Ni^0^ nanoparticles dispersed within the gel matrix, indicating that the material is loaded with nickel nanoparticles. Figure 4 shows the BET isotherm for the unloaded (a) and Ni-loaded gel. The shape of the isotherm indicates the presence of both micro- and mesopores within the gel morphological structure. The reduced surface area and lower adsorption capacity of the loaded gel compared to the unloaded one indicate that the loading of the nickel nanoparticles has partially blocked the pores in the material. The Figure 4 suggest that the nickel nanoparticles have been successfully loaded onto the porous material. The nitrogen adsorption/desorption isotherms indicate that the loading of the nickel nanoparticles resulted in a reduction in the gel pore size and surface area. The TGA results further confirm the presence of nickel nanoparticles in the material. These findings suggest that the material can be used as a support for nickel nanoparticles in various applications.

### 2.3. Removal Under Different Operating Parameters

**Figure 5:**
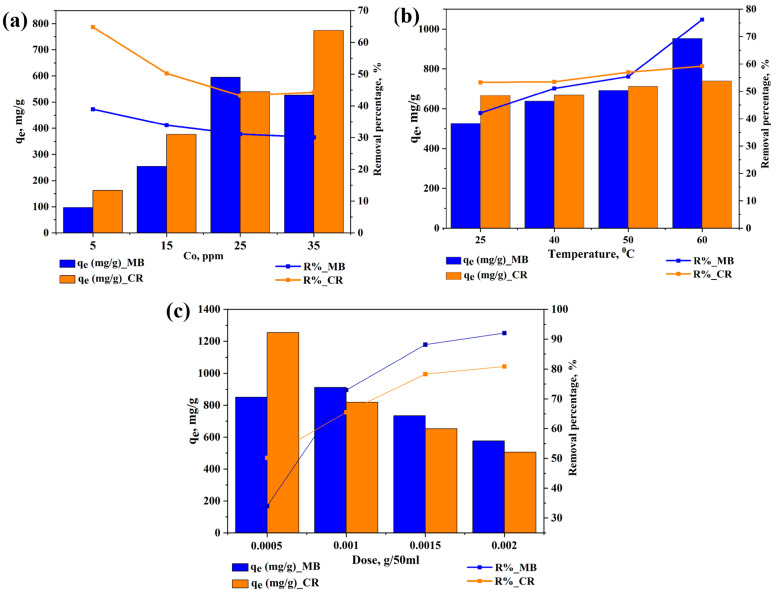
The uptake capacity and percent removal of methylene blue and Congo red at equilibrium at various initial concentrations of the dye solutions (**a**), used dose of the adsorbent (**b**), and operating temperature (**c**).

The elimination of both the MB and CR dyes from aqueous solutions was assessed using the impregnated gels across various time intervals. The kinetic profiles, plotting adsorption capacity (q) against time (t), indicated that at equilibrium, the amount of dye adsorbed (q_e_) remained constant. The studies were conducted under varying initial dye concentrations, adsorbent doses, and temperatures to determine q_e_ and the percentage removal for each condition, shown in Figure 5. Control samples with 0% loading of Ni^0^ NP were prepared and tested. However, the removal in this case showed low values compared with the high values of the loaded samples.

Figure 5a illustrates that the adsorption capacity and percentage removal are influenced by the initial concentration of the dye solution at an adsorbent dose of 1 g/L and 25 °C. It is obvious from the figure that the uptake capacity at the equilibrium of each dye increased with the increase in the initial concentration of the dye solution, since a greater mass transfer driving force resulted from higher initial concentrations [33,34]. Interestingly, in the case of the CR dye, this increase in percent removal was particularly noticeable at smaller values of the initial concentrations. However, at greater values of the solution concentrations, the dye elimination efficiency diminished due to the saturation of the gel and nanoparticle active sites, which was observed for both MB and CR. The figure also indicates that nickel nanoparticles were more effective at removing MB than CR. Figure 5b shows that as the adsorbent dose increased, the uptake decreased, likely because of the clustering of the nanoparticles, which reduced its total surface area [35]. Nonetheless, in the case of a greater dose amount of the gel, the removal efficiency was initially improved until it reached a saturation point. At a dose of 0.02 g/L and 25 ppm initial concentration at room temperature, higher doses removed approximately 90% of MB and 70% of CR.

Temperature effects were more pronounced, with both uptake and percentage removal slightly increasing with temperature (Figure 5c). This trend, characteristic of endothermic processes, is driven by an active surface and a higher number of unoccupied sites [36]. At a 60 °C temperature, 25 ppm initial concentration, and 0.02 g/L dose, removals of approximately 80% for MB and 60% for CR were achieved. The initial concentration was chosen because it reflects the typical concentration used in industrial applications.

### 2.4. Characterization of the Adsorption and Degradation Processes

**Figure 6:**
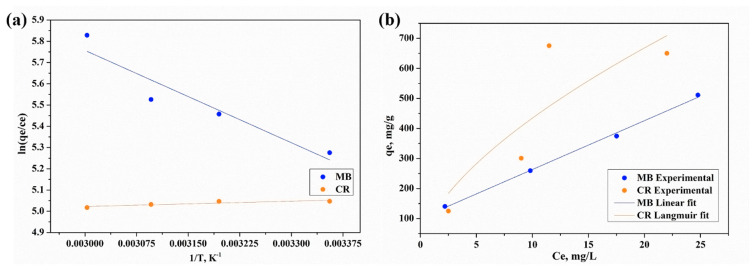
Plots of van’t Hoff (**a**) and equilibrium isotherms (**b**) for MB and CR adsorption.

Thermodynamic parameters for dye adsorption, including enthalpy (ΔH), entropy (ΔS), and Gibbs free energy (ΔG), were estimated using the van’t Hoff plot (Figure 6a) and are summarized in Table 1.

These parameters are essential for understanding the adsorption mechanism. The positive ΔH values for both methylene blue and Congo red adsorption processes indicate that these processes are endothermic. Endothermic processes involve the dissociation of adsorbates, which correlates with dye degradation in this case. Positive ΔS values further support this by indicating an increase in randomness due to dye dissociation [37]. The low ΔH values suggest that the energy associated with dye dissociation or chemisorption is relatively low.

The adsorption of Congo red is both spontaneous and favorable, as evidenced by the negative Gibbs free energy and a value for the equilibrium constant that is higher than one. Adsorption isotherms (Figure 6b) indicate that methylene blue (MB) adsorption follows a Langmuir-type isotherm (R^2^ = 0.9149), with complete coverage of the active surface (q_m_ = 1111.11 mg/g). In contrast, CR adsorption adheres to a linear isotherm (R^2^ = 0.9544), showing an incomplete surface coverage within the tested concentration range. This suggests that achieving surface saturation for CR would require significantly higher concentrations, which may not be feasible. The differing isotherm behaviors are likely due to the nature of the degradation products for each dye. CR may degrade into products that are more prone to desorption than adsorption, thus escaping from the active surface. Conversely, MB breaks down into products that can easily attach to the active surface as a result of favorable surface chemistry and alignment, leading to the observed surface saturation of the catalytic nanoparticles, which is in line with the observation that MB has a higher removal efficiency than CR.

Photocatalytic degradation involves exciting metal nanoparticles with photons to generate electron–hole pairs. Typically, this process results in harmless byproducts [38]. As an example, photodegradation of MB results in the formation of demethylated intermediates of Azure and benzenesulfonic acid [39,40,41,42]. In the case of Congo red, initial degradation involves benzene ring cleavage by nanocrystalline titanium dioxide, followed by the breakdown of the C-S, C-N, C-C, and N=N bonds. These intermediates are eventually decomposed into carbon dioxide and water [43,44].

### 2.5. Kinetics of Adsorption

**Figure 7:**
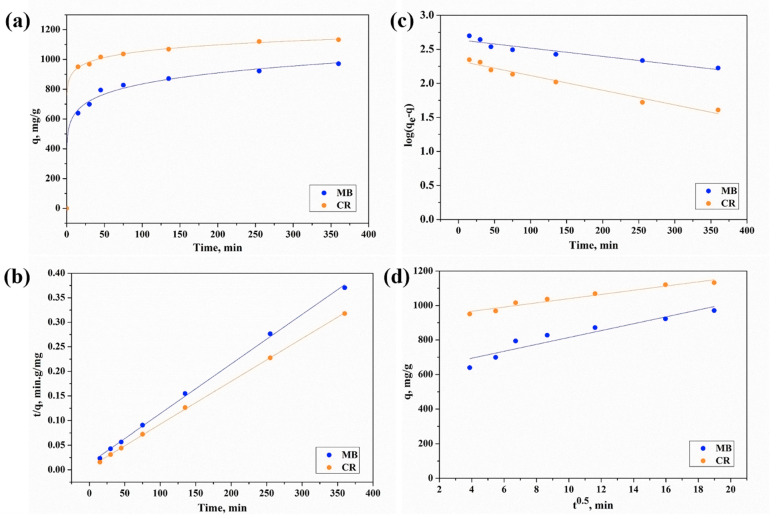
The kinetic plots of the removal of methylene blue and Congo red dyes (**a**), linear isotherms estimated using the pseudo-first-order model (**b**), linear isotherms estimated using the pseudo-second-order model (**c**), and linear isotherms estimated using the intra-particle diffusion model (**d**).

Figure 7a illustrates the kinetic removal plots for the adsorption of methylene blue and Congo red, showing that the latter has a higher removal efficiency than that of the former throughout all time intervals tested. These profiles were analyzed using the pseudo-first-order and the pseudo-second-order kinetics models to describe the adsorption kinetics. The linear isotherms shown in Figure 7b,c were used to evaluate the various parameters of the kinetics models and are detailed in Table 2. It should be noted that the correlation factor (R^2^) for each model signifies that the adsorption process is best described by the pseudo-second-order model, as it produced the highest R^2^ values and accurately predicted the equilibrium adsorption capacities (q_e_). Notably, the kinetic rate constants predicted by this model were identical for both dyes, suggesting similar rates for the adsorption process. The pseudo-second-order model implies that the adsorption process encompasses gel and pore diffusion as well as surface interactions.

To further investigate the driving forces behind the adsorption mechanism, the intra-particle diffusion model was applied, shown in Figure 7d. The linearity of these plots for MB and CR suggests that intra-particle diffusion governs adsorption.

Nevertheless, it can be concluded that pore diffusion is not the only rate-limiting step—since the plots do not intersect at the origin—and other processes, such as gel diffusion, may also play a role [18]. Additionally, the rate constant of the intra-particle diffusion model for Congo red is greater than that of methylene blue, which denotes faster diffusion kinetics. The larger constant “C” for Congo red suggests that gel diffusion plays a more significant role here than in the adsorption of methylene blue.

Comparing the above investigation for Ni-loaded gels with the previous results obtained for Co-loaded ones [28] confirms that the pseudo-second-order model most significantly represents the process kinetics for both dyes. This indicates that the surface interactions play a crucial role in the process. However, the nickel nanoparticle study demonstrated a clear association with the Langmuir isotherm for methylene blue, which suggests a complete surface coverage, while the cobalt nanoparticle study demonstrated an incomplete surface coverage for Congo red following a linear isotherm. Overall, both studies demonstrate effective dye removal but with differences in adsorbent performance, kinetic behavior, and material sustainability. In particular, the nickel nanoparticle (Ni^0^ NP) adsorbent achieved a higher removal efficiency, with up to 90% for MB and 70% for CR, especially at higher adsorbent doses. The cobalt nanoparticle (Co^0^ NP) adsorbent demonstrated a more balanced removal efficiency, with a maximum of 60% for both dyes under optimal conditions. It should be noted that both bio-based systems offer improved sustainability by reducing the risk of nanoparticle release into the environment and, therefore, focusing on both performance and environmental impact mitigation.

## 3. Conclusions

Nanoporous bio-based polymeric gels in situ impregnated with nickel nanoparticles have been explored for their efficacy in the removal of organic dyes with major health risks, including the potential to cause nausea, vomiting, and tissue necrosis. The successful development of sustainable gels loaded with nickel nanoparticles presents a promising approach for removing organic contaminants while protecting the environment. The gels demonstrated a high efficiency, achieving removal rates of up to 90% for MB and 80% for CR even at reduced concentrations, making them suitable for real-world applications. Their sustainability is enhanced by the use of bio-based polymers and the integration of nickel nanoparticles, reducing the risk of nanoparticle release into the environment. Additionally, the economic feasibility of this technology is supported by the use of readily available, cost-effective materials such as cellulose acetate, PLA, and polyurethane. This study also found that the adsorption mechanism is spontaneous despite being endothermic, indicating an oxidative catalytic degradation mechanism. The kinetic modeling confirms a pseudo-second-order reaction for both methylene blue and Congo red. Nevertheless, the intra-particle diffusion model revealed faster diffusion rates for CR, likely due to its greater desorption tendency. The thermodynamic adsorption data suggested that MB follows a Langmuir isotherm, indicating a complete surface coverage, while CR follows a linear isotherm, indicating incomplete coverage. Kinetic adsorption studies at an adsorbent dose of 0.001 g/mL and 25 °C demonstrated that the initial dye concentration affected adsorption capacity and removal percentages. Thermodynamic parameters showed that the dye adsorption process was endothermic, with positive enthalpy values of 1237.87 J/mol for methylene blue and 580.56 J/mol for Congo red. This research highlights that dye molecules undergo initial physisorption, followed by dissociative adsorption, which offers a promising framework for developing efficient, sustainable methods for treating dye-contaminated wastewater, which is a crucial step toward cleaner water sources and a healthier environment. Future research could focus on evaluating the performance of these gels under different flow rates and examining their long-term stability, while the development of scalable, cost-effective production methods would be key to their broader adoption.

## 4. Methods

### 4.1. Materials

The poly(lactic acid), a semi-crystalline biopolymer produced from renewable resources such as corn starch and sugarcane, was supplied by NatureWorks, Plymouth, Minnesota, USA. The reported molecular weight (Mn) by the supplier was around 2.50 × 10^5^ Da, and the of reported density was 1.24 g/cm^3^. Following prior approaches, thermoplastic polyurethane was synthesized in the lab [25,26]. The molecular weight of PEO, acquired from Alfa Aesar, Berlin, Germany, was 1.00 × 10^6^ Da. Alfa Aesar also supplied MB and CR dyes. Nickel chloride hexahydrate with a purity up to 97%, sodium chloride with a purity up to 99.5%, and sodium borohydride with a purity up to 97% were all purchased from Loba Chemie, New Delhi, India. Fisher Chemical Company, London, UK provided the tripolyphosphate (TPP) coagulant, the pure ethanol, *N*,*N* dimethyl-formamide, and the 1,4-dioxane solvents with a purity up to 99%. No further treatment was necessary for the purchased chemicals, which were all used as received.

### 4.2. Experimental

#### 4.2.1. Preparation of the Impregnated Polymeric Gels

**Scheme 1:**
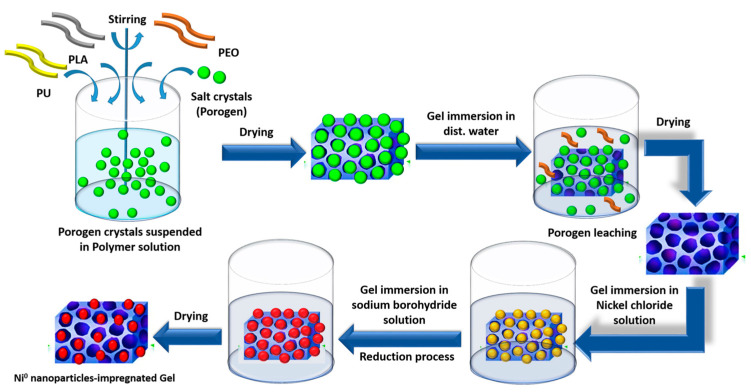
Schematic diagram for the preparation of in situ impregnated polymeric gels.

Blends of bio-based polylactic acid (PLA), biocompatible polyurethane (PU), and biodegradable polyethylene oxide (PEO), along with 10% sodium chloride (NaCl) included as porogen materials, were prepared [26]. The mixture was squeezed out in 20 mL droplets using a 22 G needle into 50 mL of 10.0% (*w*/*v*) TPP coagulant solution at pH 6.0. The solutions were maintained at room temperature for 24 min, and the hydrogel beads were freeze-dried at −45 °C overnight. The addition of the PEO enhanced the absorption of the water molecules into the gels, promoting the dissolution of the porogen salt crystals and causing the polymeric gels to swell [27]. As the porogen crystals dissolved, small pores were formed within the gels to afford the nanoporous gels. After drying, the porous gels were soaked in a 0.1 M solution of nickel chloride hexahydrate for three days on a rotary shaker to allow for metal ion impregnation. Reduction of the metal ions took place by immersing the impregnated gels in 200 mL of 0.5 M sodium borohydride on a rotary shaker for 24 h at room temperature to afford the nanoparticles. Figure S2 displays the TEM image of the resultant Ni^0^ nanoparticles. The gels were then thoroughly rinsed to ensure the complete removal of any remaining metal ions or contaminants by immersing them overnight in distilled water. The complete procedures for the preparation of the Ni^0^-impregnated gels are shown in Scheme 1.

#### 4.2.2. Characterization of the Polymeric Gels

The prepared gels underwent a series of characterization tests to evaluate their properties. A Thermo Scientific Q series instrument was used to produce the thermogravimetric analysis (TGA) of the polymeric gels. The samples’ temperature steadily increased under a nitrogen purge with a rate of 10 °C per minute up to 600 °C. The textural properties of the impregnated gels were evaluated using BET Micrometrics ASAP 2020 instrumentation. Four grams of each unloaded and loaded gel were degassed overnight at 100 °C under nitrogen gas. Additionally, scanning electron microscopy (SEM) was conducted using a SUPRA 55 LEO SEM instrument, Oberkochen, Germany, equipped with a high-resolution field emission gun scanning electron microscope.

#### 4.2.3. Removal of Methylene Blue (MB) and Congo Red (CR) Dyes

Batch contact experiments were conducted to evaluate the effectiveness of nickel nanoparticle-loaded porous gels in removing both the MB and CR dyes. The tests were performed at a pH value of four for the MB dye and three for the CR dye. These optimum pH values were identified during the screening studies for both dyes, shown in Figure S3. The experiments were carried out under varying conditions of initial dye concentration, adsorbent dosage, and temperature. A specific amount of each impregnated porous gel was soaked in a 50 mL dye solution. To prepare the methylene blue dye solutions of different concentrations, a stock solution of 100 ppm concentration was first prepared by dissolving 0.1 g (3.125 × 10^−4^ mol) of methylene blue dye in 1 L deionized water. Care was taken to store the prepared solution in a dark glass bottle, since methylene blue is light-sensitive. Dilution of the stock solution afforded the desired initial concentrations for the removal study. Similar procedures were followed for the preparation of the Congo red dye solutions by dissolving 0.1 g (1.435 × 10^−4^ mol) of Congo red in 1 L deionized water. The samples were then exposed to a 254 nm UV radiation emitted from a UV lamp source for different time intervals to initiate the photocatalysis process. At each time interval, two mL of the solution was drawn and replaced with distilled water. A UV-Vis spectrophotometer was then used to measure the equilibrium absorbance of the MB dye at 670 nm and at 500 nm for the CR dye, which was then used to calculate the corresponding equilibrium dye concentration. The adsorption capacity, denoted as q, was then calculated according to the following:

(2) $$q_{e}=\frac{\left(C_{0}-C_{e}\right)\;V}{m}$$

where

$q_{e}$: the equilibrium adsorption capacity given in mg/g,
*C*_0_: the initial dye concentration given in ppm,
$C_{e}:$ the equilibrium dye concentration given in ppm,
*V:* volume of dye solution given in L, and
*m*: the mass of nanoparticles loaded onto the porous gel given in g and accounts for 0.2% of the total mass of the gel.

Experimental results showed that the amorphous gel matrix (Figure S4) did not contribute to dye removal. Therefore, the adsorption capacity calculations were based solely on the mass of the nanoparticles. The removal efficiency (%*R*) was determined using the following equation:

(3) $$\%\;R=\frac{\left(C_{0}-C_{e}\right)\;}{C_{0}}$$

#### 4.2.4. Thermodynamic Equilibrium Evaluation

To evaluate the thermodynamic equilibrium of the adsorption process, the isotherms were fitted to various thermodynamic models, namely linear, Langmuir, and Freundlich models, according to the following:

(4) $$q_{e}={K_{e}C}_{e}$$

(5) $$q_{e}=\frac{q_{m}C_{e}K_{e}\;}{C_{e}K_{e}+1}$$

(6) $$q_{e}=K_{f}C_{e}^{1/n}$$

where *K_e_* is the equilibrium constant for adsorption, *q_m_* is the maximum adsorption capacity, and *K_f_* and *n* are Freundlich constants [28]. The slope and intercept of the van’t Hoff Equation (7) were used to estimate the enthalpy change (Δ*H*) and the entropy change (Δ*S*) for MB and CR adsorption.

(7) $$\ln\left(\frac{q_{e}}{c_{e}}\right)=-\frac{∆H}{R}\times \frac{1}{T}+\frac{∆S}{R}$$

The Gibbs free energy change (Δ*G*) was then calculated using Equation (8), and the equilibrium constant (*K_e_*) was determined using Equation (9) as follows:

(8) ∆G=∆H−T∆S

(9) $$∆G=-RTln(K_{e})$$

#### 4.2.5. Kinetics Evaluation

The kinetic uptake was estimated using various models. The pseudo-first-order model is given according to Equation (10), and the pseudo-second-order model is given according to Equation (11) [28].

(10) $$\log(q_{e}-q_{t})=\log(q_{e})-\frac{k_{1}}{2.303}\;t$$

(11) $$\frac{t}{q_{t}\;}=\frac{1}{k_{2}q_{e}^{2}}+\frac{1}{q_{e}}\;t$$

where:

*q_t_*: the uptake capacity at time *t*,
*k*_1_: pseudo-first-order rate constant, and
*k*_2_: pseudo-second-order rate constant.

The kinetic parameters were obtained by considering the slopes and intercepts of Equations (10) and (11). The Weber and Morris intra-particle model, Equation (12), is used to assess how the adsorption process is influenced by the intra-particle diffusion mechanism,

(12) $$q=k_{id}t^{0.5}+c$$

where *c* and *k_id_* are the parameters for the Weber and Morris intra-particle diffusion model.

## Figures and Tables

**Table 1:** Methylene blue and Congo red adsorption parameters.

	Δ*H* (J/mol)	Δ*S* (J/mol.K)	Δ*G* * (J/mol)	*K*_eq_ *	*R* ^2^
**MB**	1237.87	43.92	−13387.1	125.88	0.9149
**CR**	580.56	40.04	−12751.72	100.07	0.9544

* These values were calculated at 60 °C.

**Table 2:** The kinetic values determined from the various kinetic models.

Kinetic Model	Kinetic Parameters	Dye	
Pseudo-first-order	*k*_1_ (min^−1^)	MB	CR
		0.0027	0.0050
	*q*_e (calc)_ (mg/g)	436.01	214.09
	*R* ^2^	0.9056	0.9714
Pseudo-second-order	*k*_2_ (g/mg·min^−1^)	0.0001	0.0002
	*q*_e (calc)_ (mg/g)	1000	1111.11
	*R* ^2^	0.9988	0.9997
Intra-particle diffusion	*K*_id_ (mg/(g·min^0.5^)	20.003	12.216
	*C* (mg/g)	614.27	917.63
	*R* ^2^	0.9012	0.9544

## Data Availability

The original contributions presented in the study are included in the article/Supplementary Material, further inquiries can be directed to the corresponding author.

## Supplementary Materials

The following supporting information can be downloaded at: https://www.mdpi.com/article/10.3390/gels10120756/s1, Figure S1. IR spectra for the prepared gels; Figure S2. TEM images of the prepared Ni^0^ nanoparticles; Figure S3. Effect of pH on the degradation of MB (Top) and CR (Bottom) dyes; Figure S4. XRD of the unloaded (Top) and Ni^0^-loaded gels (Bottom); Table S1. Comparison of the surface area and pore size of the prepared unloaded and Ni^0^-loaded gels with other biobased polymer gels from previous studies.
