# Supplementary material for: Environmentally Friendly Nanoporous Polymeric Gels for Sustainable Wastewater Treatment
Source: Gels. 2024 Nov 22;10(12):756. doi: 10.3390/gels10120756 (PMC11675114; doi:10.3390/gels10120756)
Supplement: Supplementary file 1 [file gels-10-00756-s001.zip › gels-3308037-supplementary.pdf]

Article

Environmentally Friendly Nanoporous Polymeric Gels for Sustainable Wastewater Treatment

Tarek M. Madkour [1,*], Rasha E. Elsayed [1] and Rasha A. Azzam [2]

[1] Department of Chemistry, School of Science and Engineering, The American University in Cairo, Cairo 11835, Egypt; r.essam@aucegypt.edu
[2] Department of Chemistry, Helwan University, Ain-Helwan 11795, Egypt; (R.E.E.); rasha_azzam@science.helwan.edu.eg
* Correspondence: tarekmadkour@aucegypt.edu

Supplementary Figures

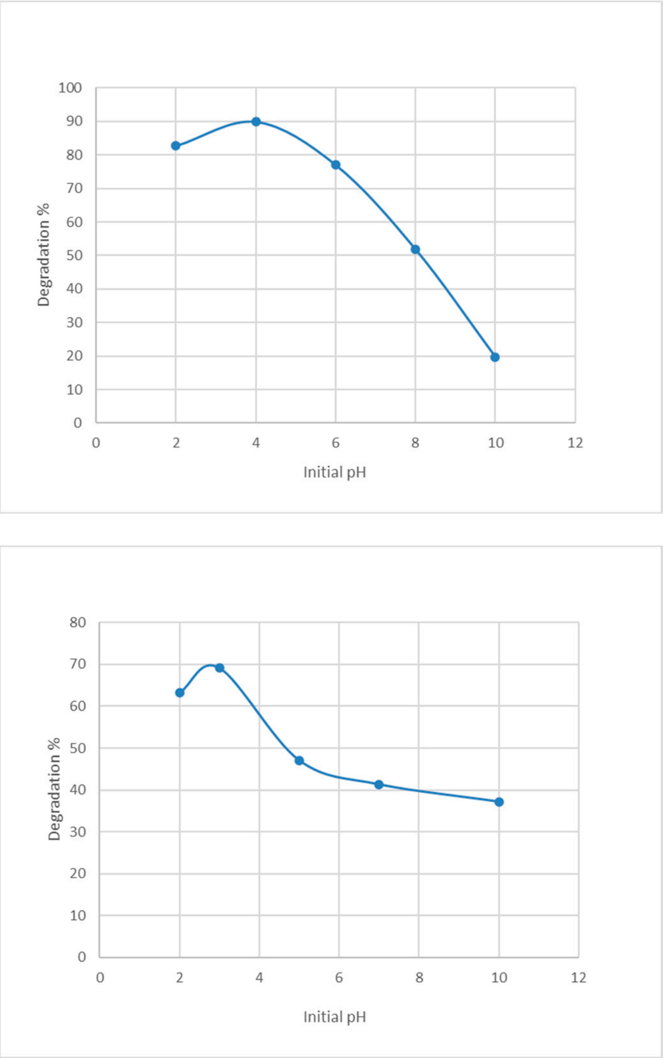

Figure S1. Effect of pH on the degradation of MB (Top) and CR (Bottom) dyes.

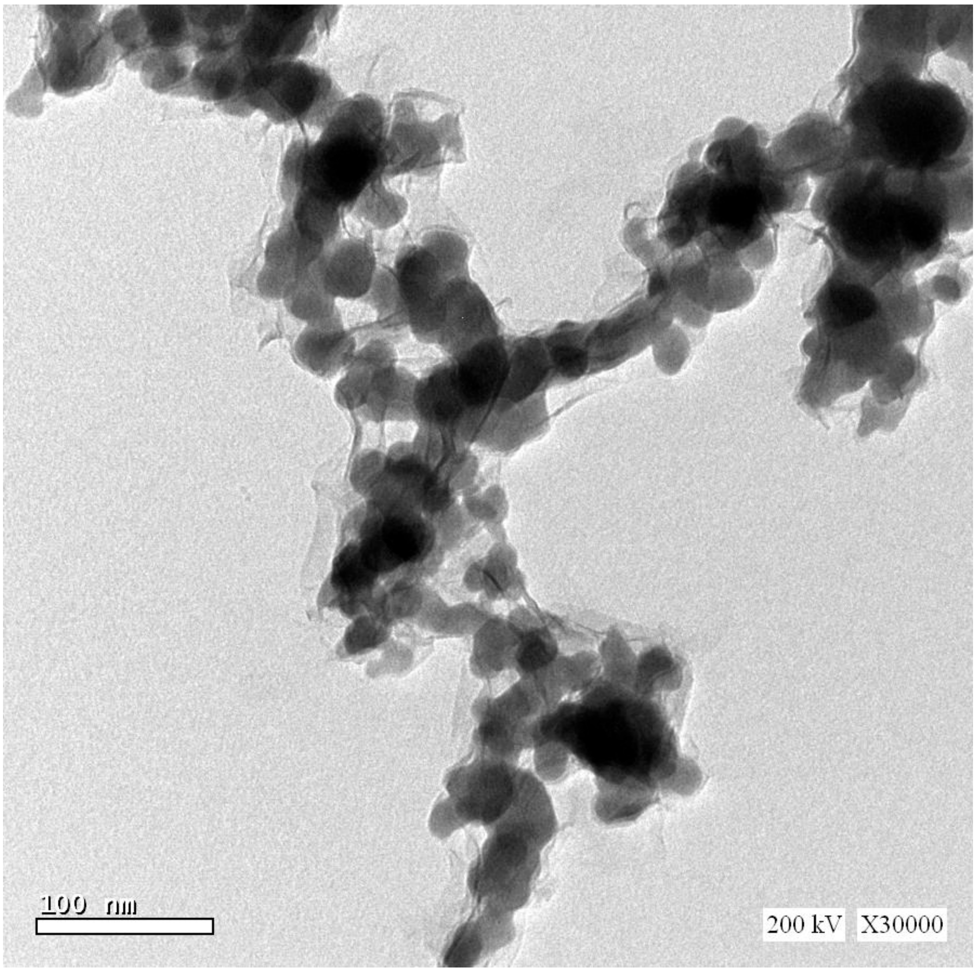

Figure S2. TEM images of the prepared Ni^0 nanoparticles.

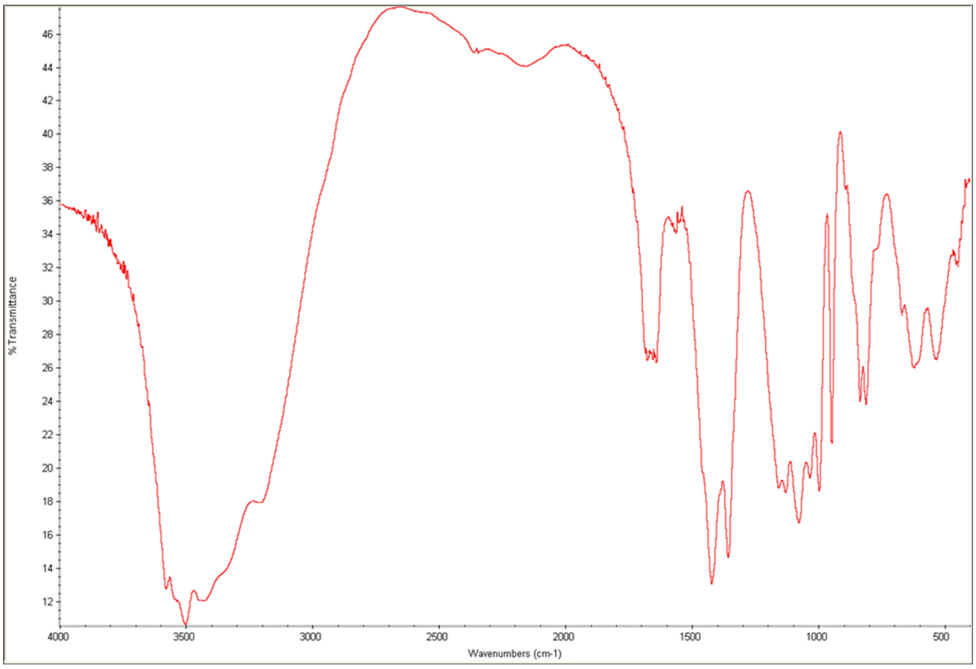

Figure S3. BET isotherms for the adsorption of nitrogen onto the unloaded gel (top panel) and the Ni-loaded gel (bottom panel).

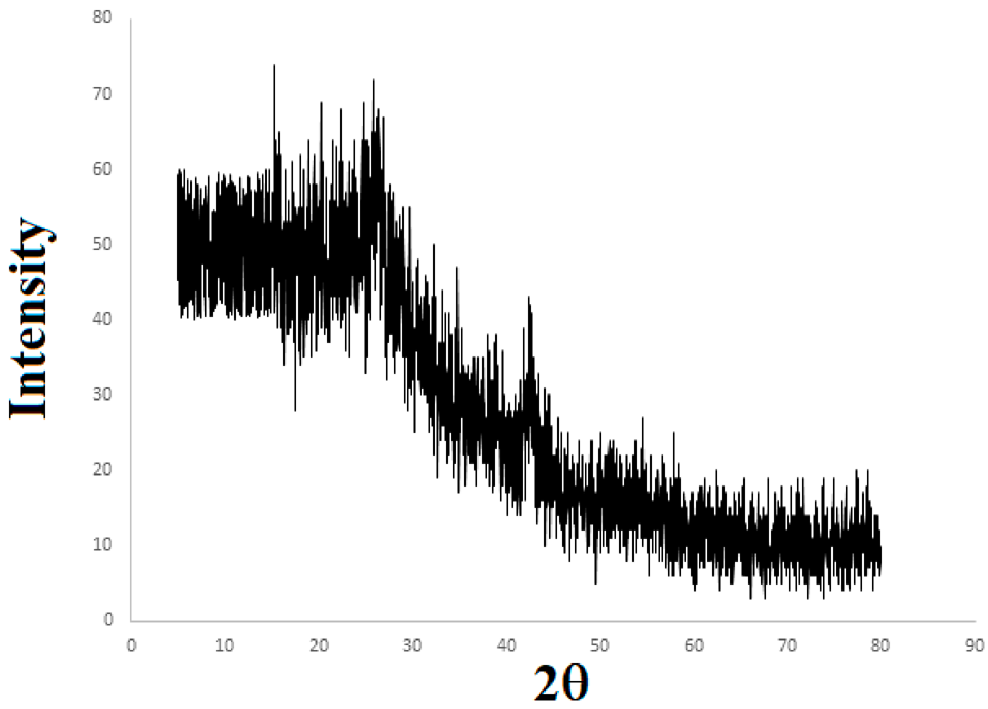

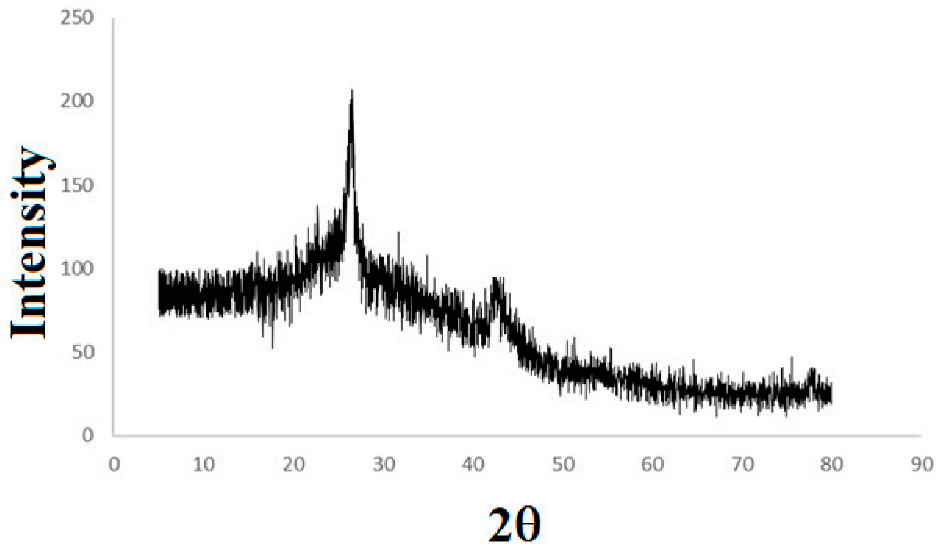

Figure S4. XRD of the unloaded (Top) and Ni^{0}-loaded gels (Bottom).

Table S1. Comparison of the surface area and pore size of the prepared unloaded and Ni^0-loaded gels with other biobased polymer gels from previous studies.

Biobased Polymer Gel System	Surface Area (m²/g)	Pore Size (nm)
Unloaded PLA/TPU/PEO gels	5.98	6.7
Impregnated PLA/TPU/PEO gels	8.5	9.8
Cellulose-Based gels[1,2]	~300	~10
Chitosan-Silica Hybrid gels[1,3]	~450	~30
Starch-Based Polymer gels[1]	~250	~50
Protein-based gels (e.g., Soy Protein) [1,3]	~200	~20

References:

1. Ganesan, K.; Budtova, T.; Ratke, L.; Gurikov, P.; Baudron, V.; Preibisch, I.; Niemeyer, P.; Smirnova, I.; Milow, B. Review on the Production of Polysaccharide Aerogel Particles. *Materials.* 2018, 11, 2144.
2. Groen, J.C.; Peffer, L.A.A.; Pérez-Ramı́rez, J. Pore Size Determination in Modified Micro- and Mesoporous Materials. Pitfalls and Limitations in Gas Adsorption Data Analysis. *Microporous Mesoporous Mater*. 2003, 60, 1–17.
3. Horvat, G.; Pantić, M.; Knez, Ž.; Novak, Z. Brief Evaluation of Pore Structure Determination for Bioaerogels. *Gels.* 2022, 8, 438.
